# Treatment of Diseases of Metabolism

**Published:** 1903-07

**Authors:** 


### Treatment of Diseases of Metabolism.

St. Just (Paris, France,) says: Diseases of metabolism include those principally, the nature of which depends upon insufficient assimilation of the ingested food stuffs, diseases, such as gout, diabetes mellitus, obesity, anemia, chlorosis, and the like. When, owing to deficient metabolism, the various parts of the human organism become affected or weakened to such an extent that they are no longer able to protect themselves against toxic effects, there will occur diseases of the liver and kidneys, digestive disturbances, tuberculosis, rheumatism and nervous affections.

The treatment of all these diseases has long since been directed toward removal of the causative agent, by proper nutrition and by adherence to ascertained diet, which was regulated according to the various pathological symptoms.

The anemics and chlorotics were given strengthening food; the adipose were prohibited from eating fatty meats; the diabetics were not allowed to eat sugar and related substances, such as bread; the gouty subjects were to abstain from too much meat, because the latter favored the formation of toxins, owing to its abundance of nucleated cells; the neurasthenics had to do without blood-irritating substances, such as bouillon and certain kinds of meat, beer, coffee, and the like, to avoid further irritation of their overtaxed nerves, and so forth.

Although such abstinence cures are most disagreeable to the patient, it cannot be denied that they generally cause a rapid diminution of the morbid symptoms. But the improvement does not last long. As soon as the patient returns to his old mode of living, his disease reappears in its fullest extent. Permanent cures were accomplished but rarely, even when the patients adhered to the prescribed diet for months and years.

We find the explanation of these important facts, if we consider the real causative agents of all these affections. According to the kind of disease, various portions of the human organism have lost their ability to properly assimilate the ingested food, and this causes, in some of the above named diseases, a deficiency of such substances as are necessary for the proper thriving of the human organism, as in the anemic and in the chlorotic. In other affections, there remain in the human organism unassimilated, injurious portions of the ingested food stuffs, which then find expression as gout, diabetes mellitus, neurasthenia, and the like. Briefly, the cells of the human organism have lost their energy properly to functionate.

This energy, however, is imparted to the cells solely by the oxygen, of which ample quantities are present in the air, and which is inhaled through the lungs. The just recognition of this fact was the cause of making such patients take plenty of outdoor exercise, thus materially increasing the capacity of the blood and of the lungs to absorb oxygen.

Under the present day industrial conditions, however, many of the people are not able to adequately breathe in the open air, hence this capacity of the lungs to absorb oxygen is only a limited one. Dr. Friedrich Elias, a chemist of Berlin, has succeeded in producing a true magnesium dioxide, which contains abundant quantities of diffusible oxygen, and which enables the practitioner to administer any desired quantity of this important element of life. Besides, this new chemical agent, which is called biogen, contains oxygen in active form, and it can, therefore, exert a much greater energy than the frequently contaminated, inactive oxygen of the air. This plentiful administration of oxygen imparts increased vigor to the cells of the human organism; they regain their former ability to assimilate ingested substances and to properly functionate again. Not only do the external morbid symptoms disappear as a result of the use of biogen ($Mg O_2$), but a thorough and permanent cure is accomplished in all cases of oxygen hunger. In failing compensation of heart disease and all attacks of dyspnea, however caused, biogen parts freely with its high percentage of oxygen, and supplements pulmonary respiration. In emergencies, large doses may be given and maintained with impunity and with no consequent deleterious effect. In combined heart and kidney lesions, in all conditions of defective elimination, rheumatism and gout, prompt relief is secured from the increased supply of oxygen which the patient receives, and this new agent, biogen, has a distinct and pronounced value. In cases of pneumonia, asthma, tuberculosis and diabetes mellitus, it will be found specially effective.

